# The Heterocellular Emergence of Colorectal Cancer

**DOI:** 10.1016/j.trecan.2016.12.004

**Published:** 2017-02

**Authors:** Christopher J. Tape

**Affiliations:** 1 The Institute of Cancer Research, 237 Fulham Road, London SW3 6JB, UK

## Abstract

Tissues contain multiple different cell types and can be considered to be heterocellular systems. Signaling between different cells allows tissues to achieve phenotypes that no cell type can achieve in isolation. Such emergent tissue-level phenotypes can be said to ‘supervene upon’ heterocellular signaling. It is proposed here that cancer is also an emergent phenotype that supervenes upon heterocellular signaling. Using colorectal cancer (CRC) as an example, I review how heterotypic cells differentially communicate to support emergent malignancy. Studying tumors as integrated heterocellular systems – rather than as solitary expansions of mutated cells – may reveal novel ways to treat cancer.

## Trends

Solid tumors contain multiple heterotypic cell types and should be considered to be heterocellular systems.

Heterotypic cell types can differentially process signals. When different cells interact (via heterocellular signaling) they expand their collective signal-processing potential to achieve emergent heterocellular phenotypes.

Cancer is an emergent phenotype that supervenes upon heterocellular signaling.

Perturbing heterocellular signaling can destabilize malignant systems as treatments for cancer.

## Cancer as an Emergent Heterocellular Phenotype

Metazoan tissues are composed of multiple cell types (e.g., epithelial and mesenchymal cells, leukocytes) [1] and can be thought of as **heterocellular systems** (see Glossary) [2]. For example, consider the mammalian intestine. Healthy intestinal tissue is a heterocellular system wherein several different cell types collaborate to form a functional organ. Notably, epithelial **enterocytes** control nutrient uptake [3], whereas mesenchymal fibroblasts support epithelial renewal [4], and tissue-resident lymphocytes and myeloid cells patrol against infection [5].

**Figure 1:**
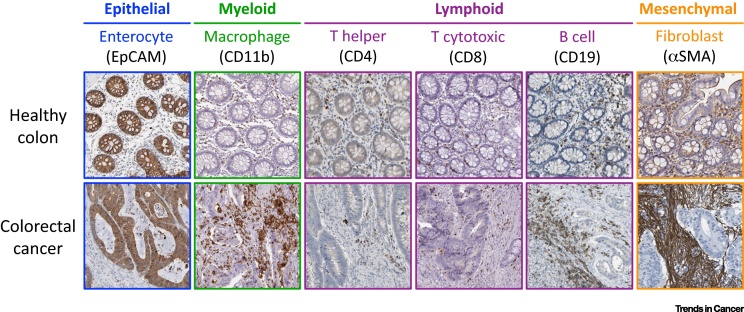
Colorectal Cancer Is a Heterocellular System. Healthy colon and colorectal cancer (CRC) immunohistochemistry sections (from the Protein Atlas, www.proteinatlas.org) [83] illustrate the explicit heterocellularity of intestinal tissue. Both healthy and CRC tissue contain epithelial cells (EpCAM^+^), myeloid macrophages (CD11b^+^), T helper lymphocytes (CD4^+^), T cytotoxic lymphocytes (CD8^+^), B lymphocytes (CD19^+^), and mesenchymal fibroblasts (αSMA^+^).

Tumors also comprise multiple heterotypic cell types. For example, similarly to the healthy colon, colorectal cancer (CRC) tumors contain epithelial cells, mesenchymal fibroblasts, myeloid cells, and lymphocytes [6]. Like most solid tumors, CRC tumors are therefore not merely **homocellular systems** or pools of epithelial cells but are integrated heterocellular systems (Figure 1).

**Figure 2:**
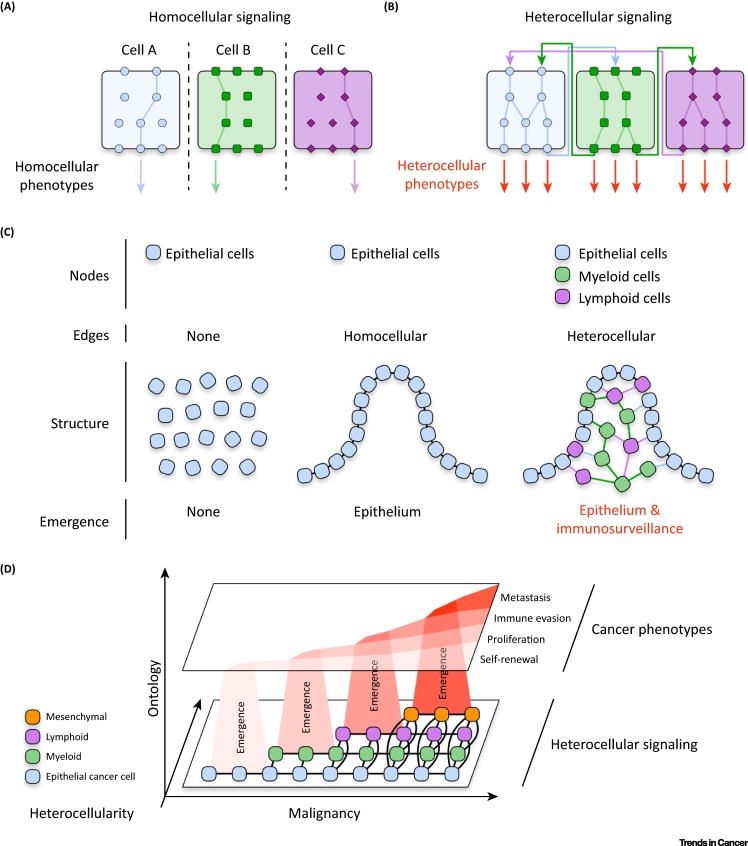
Key Figure: Cancer Phenotypes Supervene Upon Heterocellular Signaling.

Heterotypic cells process and interpret signals completely differently 7, 8. This cell-specific **homocellular signaling** enables differentiated cells to achieve distinct phenotypes (Figure 2A, Key Figure). When multiple cell types are combined, **heterocellular signaling** between cells can take place [2]. Because each cell type has a different signal-processing capacity, heterocellular signaling can engage signaling pathways that each cell type cannot activate autonomously [9]. This signaling expansion enables heterocellular systems to achieve phenotypes beyond those of each cell type in isolation (Figure 2B). For example, myeloid dendritic cells can use major histocompatibility complex (MHC) class-II signal processing to present antigens to lymphoid cytotoxic T cells. In turn, activated T cells can use their unique signaling to launch a cytotoxic immune response against the antigen. Together, the two cell types can achieve adaptive immunity. In isolation they cannot.

When several interacting constituents achieve an output beyond the sum of their inputs, an **emergent system** is formed [10]. Such a system requires two core elements: (i) constituent ‘nodes’ and (ii) interacting ‘edges’ connecting the nodes. When considering tissue, ‘nodes’ can be thought of as cells and ‘edges’ as intercellular signals. For example, several epithelial cells (nodes) can interact via adherens junctions (edges) to form an emergent homocellular epithelium – whereas non-interacting epithelial cells cannot. One way to expand the output of an emergent system is to increase the ‘diversity’ between nodes. For example, while a homocellular network of interacting epithelial cells can produce an epithelium, a heterocellular system of interacting epithelia, myeloid cells, and lymphocytes can produce epithelium with adaptive immunological surveillance. When different cell types interact to produce tissue-level phenotypes, we can say that **heterocellular emergence** has occurred (Figure 2C). Heterocellular emergence requires: (i) cell ‘nodes’, (ii) intercellular signaling ‘edges’, and (iii) heterotypic ‘diversity’ between cell nodes. Because heterocellular emergence requires different cell types to communicate with each other, tissue-level phenotypes **supervene** upon heterocellular signaling.

Similarly to healthy tissues, tumors are integrated heterocellular systems that achieve complex phenotypes (e.g., metastasis, immune evasion). We can therefore suppose that malignant phenotypes also supervene upon heterocellular signaling (Figure 2D). Anecdotal examples of heterocellular signaling are widely reported across the **tumor microenvironment** of many different cancers 11, 12. Nevertheless, despite the explicit heterocellularity of tumors, most cancers are still studied as homocellular pools of cancer cells. For example, CRC has long been considered to be a disease of mutated epithelial tumor cells. As such, most attempts to treat CRC focus on inhibiting epithelial cells directly – with little regard to the tumor microenvironment they occupy in patients. However, recent studies have revealed that CRC tumors are highly heterocellular, and increased heterocellularity leads to poorer survival 13, 14, 15, 16. To treat cancer more effectively, we must understand how heterotypic (diverse) cells (nodes) interact (edges) to achieve malignant phenotypes. Using CRC as an example, I discuss here how heterotypic cells interact to form an emergent malignant system – and how this integrated topology can be targeted to treat cancer.

## Homo- versus Heterocellular Signaling in CRC

The healthy colon is lined by a single sheet of continually renewing epithelial cells. This physical barrier separates the intestinal lumen (containing nutrients, commensal microflora, and pathogens) from subepithelial tissue. The healthy colonic epithelium is organized into a repetitive crypt structure in which pluripotent intestinal epithelial stem cells either self-renew at the crypt base or differentiate into absorptive enterocytes and goblet cells along the crypt [3].

Colonic malignancy is driven by **oncogenic mutations** in epithelial cells. Common CRC mutations include loss of APC function (∼80%), loss of p53 (TP53, ∼60%), and hyperactivation of KRAS (∼40%) [17]. These mutations occur as a ‘big bang’ event in a limited subset of epithelial cells [18] and seem to drive malignant phenotypes by rewiring **cell-autonomous signaling** in epithelial cells. For example, loss of APC hyperactivates β-catenin signaling [19], whereas *KRAS* point mutations hyperactivate the RAF–MEK–MAPK cascade [20]. This deregulated cell-autonomous signaling promotes epithelial proliferation and self-renewal. Thus, when viewed from a homocellular perspective, emergent malignant phenotypes appear to supervene upon deregulated cell-autonomous epithelial signaling.

**Figure 3:**
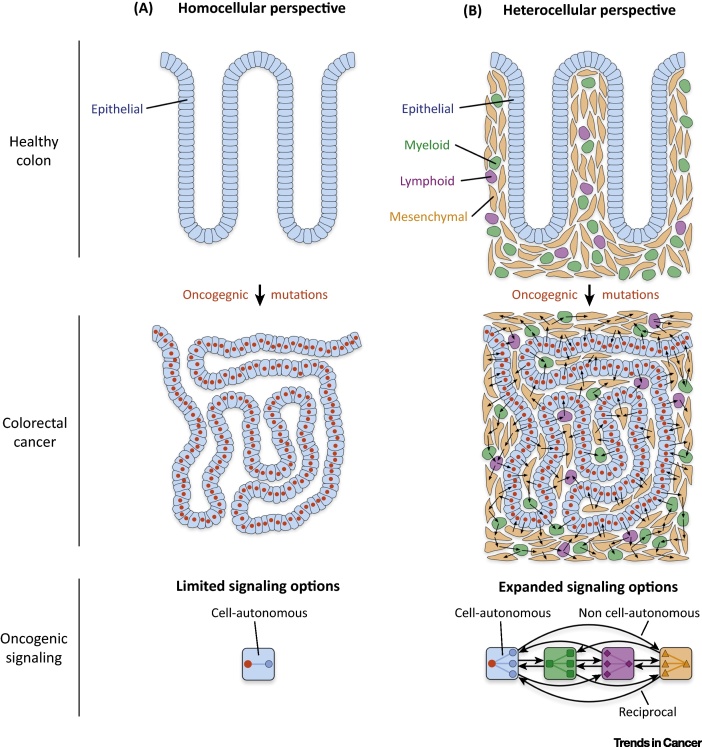
Homo- and Heterocellular Oncogenic Signaling. (A) Similarly to many cancers, colorectal cancer (CRC) is often viewed as a homocellular system (i.e., only epithelial cancer cells are studied). When viewed from this homocellular perspective, epithelial oncogenic mutations can only drive malignant phenotypes through cell-autonomous signaling (e.g., loss of APC function hyperactivates epithelial β-catenin signaling). (B) However, because CRC tumors are explicitly heterocellular (Figure 1), oncogenic mutations can explore a wide range of heterocellular signaling options. This heterocellular diversity increases oncogenic signaling opportunities (e.g., cell-autonomous, **non cell-autonomous**, and reciprocal signaling) – enabling tumors to achieve complex emergent malignant phenotypes.

However, this homocellular view ignores the explicit heterocellularity of metazoan biology. Both in healthy and diseased tissue, colonic epithelia always interact with intestinal mesenchymal cells, lymphocytes, and myeloid cells. Each of these heterotypic cell types processes signals differently from epithelial cells, and can subsequently facilitate unique heterocellular phenotypes. Consequently, an emergent model of cancer must include heterocellular signaling between all cell types in the tumor microenvironment (Figure 3).

## A Heterocellular Environment

Nestled beneath the healthy colon epithelia is the **lamina propria**. This heterocellular structure contains multiple leukocyte lineages and mesenchymal fibroblasts [4]. The healthy intestine is entirely reliant on heterocellular signaling between epithelial cells and fibroblasts [21]. Notably, fibroblasts are maintained by epithelial Hedgehog signaling [22] and, in turn, fibroblasts secrete Wnt ligands and BMP antagonists to support the epithelial stem cell niche 4, 23. Neither cell type alone is sufficient to maintain intestinal homeostasis – but together they perpetually achieve heterocellular emergence.

Given the intimate relationship between epithelial and mesenchymal cells in the healthy colon, it is perhaps unsurprising that fibroblasts are also involved in colonic malignancy. When colonic epithelial cells acquire oncogenic mutations (such as loss of APC), proximal fibroblasts proliferate [24]. This is accompanied by increased expression of the myofibroblast marker αSMA and secretion of matrix metalloproteinases [25]. Increased myofibroblast abundance drives epithelial liver metastasis and predicts CRC recurrence after surgery 26, 27. Increased mesenchyme-derived desmoplasia also correlates with poor prognosis [28] and metastatic recurrence in CRC 29, 30. Collectively, mesenchymal fibroblasts are a major marker of poor prognosis in CRC 13, 14.

Because oncogenic mutations only occur in epithelial cells, malignant mesenchymal phenotypes must supervene upon heterocellular interactions. To achieve this, oncogenic epithelial cells need to differentially communicate with fibroblasts (or, to use network parlance, ‘malignant edges’ must form between epithelial and mesenchymal nodes). Although little is known regarding specific heterocellular interactions in CRC, several studies have revealed that colonic epithelial cells can activate fibroblasts via TGF-β 25, 31 and poor-prognosis mesenchymal CRC subtypes display TGF-β-induced epithelial expression signatures. In addition to TGF-β, epithelial IL-33 also activates fibroblasts [32]. Reciprocally, these activated fibroblasts then promote the proliferation and migration of intestinal epithelial cells via amphiregulin, HGF, FGF, and Wnt signaling 33, 34, 35. The pro-malignant role of mesenchymal cells is not limited to the primary tumor because liver fibroblasts also support epithelial cancer cells in the metastatic niche [36]. These observations support the hypothesis that mutated epithelial cells use the alternative signal-processing capacity of mesenchymal cells to achieve emergent malignant phenotypes in CRC.

While focusing on epithelial–mesenchymal heterocellular interactions provides more insight than studying epithelial cells alone, mesenchymal fibroblasts are only one of many non-epithelial cell types in CRC tumors. For a more complete heterocellular perspective, interactions between epithelial cells, fibroblasts, and tissue-resident leukocytes cells must also be considered.

## Immunological Diversity

The gastrointestinal tract is a major site of host–pathogen interaction. As a result, healthy intestinal tissue is richly populated with leukocytes that patrol against infection [5]. Macrophages are the most profuse leukocyte in the colon [5], and their abundance increases further during CRC tumorigenesis [24]. Similarly to myofibroblast expansion, heterocellular signaling also drives the recruitment of myeloid macrophages. For example, epithelial cancer cells produce IL-10 [37] and CCL2 [38] to recruit tumor-associated macrophages (TAMs). In turn, TAMs express SIRPα, which can reciprocally increase epithelial migration via CD47 [37]. TAMs can also secrete IL-6 to activate epithelial JAK–STAT signaling to promote proliferation and invasion 39, 40, 41, 42. Moreover, TAMs play a major role in processing CRC extracellular matrix (ECM), and can also regulate collagen production by fibroblasts [43]. These emergent heterocellular phenotypes supervene upon **reciprocal signaling** between epithelial cells, mesenchymal fibroblasts, and TAMs.

In addition to myeloid TAMs, multiple lymphocytes infiltrate into the lamina propria during CRC tumorigenesis 24, 44. Adaptive immunity is regulated by heterocellular interactions in both homeostatic tissue and cancer. For example, mutations in epithelial CRC cells produce unique neo-antigens that can be recognized by tumor-infiltrating T cells [45] (including oncogenic KRAS specifically [46]). As a result, CRC tumors with increased lymphocytic reaction have a better prognosis 47, 48, 49, 50. This proinflammatory response can be regulated by TAMs and prime T cells towards an antitumor response [51]. Conversely, suppressed T lymphocyte cytotoxicity facilitates epithelial cancer cell metastasis [52]. The role of B lymphocytes is less clear – although recent evidence suggests they may be tumor-suppressive [53]. Thus, both in the primary tumor and in distant metastasis, tumor immunogenicity supervenes upon heterocellular interactions between epithelial cancer cells, myeloid cells, and lymphocytes.

Although typically considered as hubs for signal processing and ECM production, it is becoming increasingly clear that mesenchymal fibroblasts play a major role in regulating both innate and adaptive immune responses. For example, myofibroblast abundance correlates with the increased presence of cytotoxic T cells in CRC [27]. This can result from fibroblast Toll-like receptor (TLR) activation, which upregulates proinflammatory cytokines that can recruit lymphocytes and myeloid cells into a tumor [54]. Intestinal fibroblasts can also act as non-professional antigen-presenting cells (APCs) to the adaptive immune system 55, 56. This suggests that fibroblasts may interact with lymphocytes in the tumor microenvironment to support immunosurveillance. However, intestinal myofibroblasts can also suppress the proliferation of helper T cells [57], induce colonic regulatory T cells (Tregs) [58], and secrete anti-inflammatory IL-11 59, 60 – and can be considered to be immunosuppressive [61]. As a result, the exact role of fibroblasts in regulating CRC immunogenicity is still under investigation.

**Figure 4:**
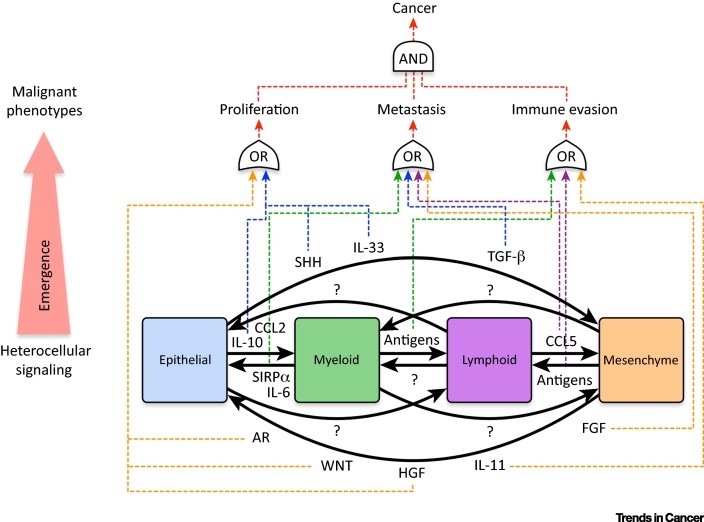
Established Heterocellular Signaling In Colorectal Cancer (CRC). Known heterocellular interactions that drive emergent phenotypes in CRC. While much progress has been made, our understanding of heterocellular signaling in cancer is vastly incomplete. For example, in CRC, epithelial–lymphoid and myeloid–stromal interactions are poorly characterized. Moreover, how oncogenic mutations in epithelial cells (e.g., loss of APC, KRAS^G12D^, loss of p53) regulate these heterocellular signaling pathways is currently unknown. Abbreviations: AR, amphiregulin; CCL, chemokine (C-C motif) ligand; FGF, fibroblast growth factor; HGF, hepatocyte growth factor; IL, interleukin; SIRP, signal regulatory protein; SHH, Sonic hedgehog; TGF, transforming growth factor.

Cancer immunogenicity is a complex heterocellular process. This emergent phenotype can only be achieved by a heterocellular system composed of epithelial, myeloid, lymphoid, and mesenchymal cells. No individual cell type possesses the signal-processing capacity to achieve such emergence on its own. Given the important role of both myofibroblast activation and cancer immunogenicity in CRC prognosis, this provides further evidence that tumors can only be understood as interconnected heterocellular systems (Figure 4).

## Targeting Heterocellular Emergence

If malignant phenotypes supervene upon heterocellular signaling, then perturbing heterocellular nodes and/or edges could present a powerful approach to treat cancer. Although our knowledge of CRC as a heterocellular system is deeply incomplete, attempts to disrupt heterocellular signaling are already underway.

Several studies have focused on disrupting epithelial–mesenchymal interactions [6]. Notably, perturbation of FAP^+^ fibroblasts can inhibit CRC progression [62], inhibition of mesenchymal IL-33 signaling blocks epithelial CRC proliferation [63], and inhibition of mesenchymal TGF-βR1 signaling disrupts epithelial CRC metastases [14]. In addition to epithelial–mesenchymal interactions, disruption of myeloid and lymphoid interactions in CRC is an area of intense interest [64]. For example, inhibition of CCL5 signaling between T lymphocytes and macrophages can reduce epithelial CRC metastasis [65]. This finding suggests that interactions between discrete myeloid and lymphocyte lineages can regulate the emergent malignant phenotype of epithelial cells – further emphasizing the interconnected nature of heterocellular emergence.

Although CRC tumors do not demonstrate dramatic responses to immune-checkpoint inhibitors (as observed for melanoma and lung cancer [66]), disrupting epithelial–lymphocyte interactions has shown some promise in CRC patients. For example, **mismatch-repair (MMR)-deficient** (also known as microsatellite instable, MSI) epithelial CRC cells express higher levels of PD-L1, have a higher neo-antigen load [67], and subsequently respond better to T lymphocyte PD-1 blockade [68]. However, because only 15% of patients are MMR-deficient, much is still unknown regarding epithelial–immune interactions in CRC. Interestingly, high myeloid infiltrate (as seen in CRC) can suppress checkpoint inhibitor efficacy, and inhibition of myeloid-specific PI3Kγ signaling improves T cell cytotoxicity towards epithelial cancer cells [69]. These results suggest combined inhibition of cell-specific signaling nodes such as myeloid PI3Kγ, lymphocyte PD-1, and mesenchymal TGF-βR1 could provide a powerful way to treat CRC as an interconnected heterocellular system.

Collectively, these nascent observations suggest that coordinated inhibition of cell-specific mesenchymal, myeloid, and lymphoid signaling nodes can disrupt malignant emergence in CRC. However, experience from other cancer types should prompt caution. For example, when inhibitors of mesenchymal SMO were used to treat pancreatic ductal adenocarcinoma (PDA), patients actually performed worse [70]. This may be because, in addition to supporting pro-tumor fibroblasts, SMO signaling is also essential for antitumor T lymphocyte activity [71]. By focusing only on epithelial–mesenchymal signaling, pro-tumor consequences of the drug in other cell types were overlooked. Future efforts to target heterocellular signaling need to consider the response of all cell types in a tumor – and not only of those thought to contain the hypothesized drug target.

## Concluding Remarks

The heterocellularity of metazoan life allows tumors to explore a broad range of signaling options. This expanded signaling potential enables cancer to achieve heterocellular phenotypes that no cell type can accomplish in isolation. As a result, tumors cannot be understood by monitoring cancer cells alone – cancer must be studied as an integrated heterocellular system.

Our current knowledge of the tumor microenvironment comes from assembling anecdotal heterocellular signaling events. However, to study cancer as a truly integrated heterocellular system, we eventually need to measure not only epithelial–mesenchymal interactions, or how myeloid cells signal to lymphocytes – but how all the cell types simultaneously collaborate to drive tumors (see Outstanding Questions). Although extremely challenging, such integrative studies are now theoretically possible. Biomimetic (e.g., organoid co-cultures) and genetically engineered mouse models provide solid experimental systems to observe heterocellular emergence. These model systems can then be interrogated using new cell-specific signaling technologies that enable researchers to measure heterocellular signaling at the systems-biology level (reviewed in [2]). Methods such as heterocellular multivariate proteomics 72, 73 and single-cell mass-cytometry 74, 75 can provide cell-specific signaling data from complex heterocellular models. Advances in dimensional reduction 76, 77 and mutual information analysis [78] can then compute cell-specific signaling data into predictive models of heterocellular phenotypes. By combining genetically engineered metazoan models, cell-specific signaling technology, and high-dimensional computational analysis, future researchers will be able to study tumors as integrated heterocellular signaling systems.

Previous research suggests that heterotypic (diverse) cells (nodes) interact (signal) to achieve complex heterocellular emergence. By treating cancers as integrated heterocellular systems – rather than as simple homocellular expansions of cancer cells – new therapeutic opportunities are already being discovered. Future attempts to treat cancer will likely involve disrupting novel heterocellular interactions across epithelial, mesenchymal, myeloid, and lymphoid cells. Because comparable heterocellular interactions have been reported across many solid tumors – including breast [79], pancreatic [80], prostate [81], and ovarian cancers [82] – heterocellular emergence is likely to be a universal feature of cancer.

Outstanding Questions How do oncogenic mutations (e.g., loss of APC, KRAS^G12D^, loss of p53) regulate heterocellular signaling?

Do heterocellular signals converge on common pathways – or does each patient have a unique heterocellular signaling structure that can be targeted?

Are heterocellular signaling pathways distinct across different cancer types – or do all cancers employ similar heterocellular signaling networks?

How does heterocellular signaling in the primary tumor differ from that in distant metastases?

## Glossary

**Cell-autonomous signaling**: mutation-driven signaling within the mutated cell.
**Emergent system**: emergence is a process in which complex higher-order features are formed from interactions between multiple lower-order entities.
**Enterocyte**: differentiated intestinal epithelial cells that absorb nutrients from the intestinal lumen.
**Heterocellular emergence**: a process where complex phenotypes are achieved through interactions between different cell types (e.g., adaptive immunity).
**Heterocellular signaling**: communication between different cell types that each have their own unique signal processing capacity. This allows each cell type to expand its signaling options.
**Heterocellular system**: a population of cells composed of multiple differentiated cell types (e.g., metazoan tissue).
**Homocellular signaling**: communication between cell types that each have the same signal-processing capacity (e.g., epithelial–epithelial cell-junction signaling).
**Homocellular system**: a population of cells composed of a single differentiated cell type (e.g., epithelium).
**Lamina propria**: sub-epithelial intestinal structure containing fibroblasts and leukocytes.
**Mismatch repair (MMR)-deficient**: a subset of CRC cells with errors in MMR DNA repair machinery.
**Non cell-autonomous signaling**: deregulated signaling in a non-mutated cell driven by mutations in a local mutated cell.
**Oncogenic mutation**: a mutation that drives malignant phenotypes.
**Reciprocal signaling**: deregulated signaling in a mutated cancer cell as a result of a non cell-autonomous signal returning from a non-mutated cell.
**Supervene**: an ontological relationship whereby higher-level properties of an emergent system depend on lower-level entities.
**Tumor microenvironment**: all cells, matrix, and nutrients in the vicinity of a tumor.
